# Evaluating value mediation in patients with chronic low-back pain using virtual reality: contributions for empirical research in Value Sensitive Design

**DOI:** 10.1007/s12553-022-00671-w

**Published:** 2022-04-29

**Authors:** Merlijn Smits, Harry van Goor, Jan-Willem Kallewaard, Peter-Paul Verbeek, Geke D.S. Ludden

**Affiliations:** 1 Radboud Institute for Health Sciences, Department of Surgery, Radboud university medical center, 116, Geert Grooteplein Zuid 10, P.O. Box 9101, 6525 GA Nijmegen, The Netherlands; 2 Department of Anaesthesiology, Rijnstate Hospital, Wagnerlaan 55, 6800 TA Arnhem, The Netherlands; 3 Faculty of Behavioural, Management and Social Sciences, University of Twente, Drienerlolaan 5, 7522 NB Enschede, The Netherlands; 4 Faculty of Engineering Technology, University of Twente, Drienerlolaan 5, 7522 NB Enschede, The Netherlands

**Keywords:** Virtual reality, Chronic Pain, Empirical research

## Abstract

Value Sensitive Design (VSD) is the most well-known method to consider values in design. It consists of three iterative phases of investigation: conceptual, empirical, and technical. Although the approach is promising, the role of empirical research remains unclear. We address two opportunities for extending the role of empirical research in VSD. First, we argue that empirical research enables us to identify values in context. Second, we explain that empirical research enables us to anticipate how technology mediates the values of users. We make our point by means of an empirical study in a real-life controlled experimental context into the value mediation of virtual reality (VR) in patients with chronic low-back pain. Using value-oriented semi-structured interviews with twenty patients, we first analyze what values these patients consider important, and how the values are experienced. The second set of interviews held after all patients used VR four weeks at home, aims to provide insight into value changes as mediated by VR. We end the article by a comparison of our empirical results with previous, often speculative, literature into values in VR. We show that empirical research benefits the VSD process by providing in-depth insight into the effects of context and technology on values and the ability to translate these insights into recommendations for more responsible design and implementation of the technology.

**Supplementary information:**

The online version contains supplementary material available at 10.1007/s12553-022-00671-w.

Value Sensitive Design (VSD) is the most well-known method considering human values in technology design [1]. Values are “lasting convictions or matters that people feel should be strived for in general and not just for themselves to be able to lead a good life or realize a good society” [ 2, p.27]. VSD consists of three phases of investigation: conceptual, empirical, and technical. The first, conceptual investigation, is aimed at the identification of users (also called ‘stakeholders’) and the values they consider important in technology design [3]. In the empirical investigation after this, users are empirically studied to understand how the novel technology affects their value experiences. The technical investigation, to conclude, is aimed at how the technology-in-design could embody the identified values via a study of similar technologies [4–6].

Considering human values in technology design is an important endeavor. Although VSD is a promising approach, it has also received critique on various elements of its framework. For example, several authors have stressed the need for an ethical framework to augment the VSD framework [6–8]. Other authors addressed the need for tools on user identification, and clarity on the role of users in the process [6, 9, 10]. Another frequent critique of VSD relates to the claim that values are ‘universal’ [3, 11, 12]. In this paper, we particularly reflect on the role of empirical research in VSD.

Based on a systematic review of papers on VSD over the past twenty years, Winkler and Spiekermann [1] conclude that VSD misses the hands-on tools that designers need to consider values in technology design. Especially the role of empirical research remains unclear [13]. Empirical research is conducted during the phase ‘empirical investigation’. In this phase, empirical research should provide insight into how a technology affects user values. Although no order of phases is prescribed, the empirical investigation generally follows after the ‘conceptual investigation’ in which values are identified, and precedes the ‘technical investigation’ in which the actual embodiment of values takes place and the design is made [1].

We argue for the need for a greater role for empirical research in VSD to (1) contribute to value identification, and (2) anticipate value mediation once a technology is implemented. First, as Dantec and Poole [13] argued, VSD seems to prioritize known values over value discovery. Instead of studying empirically what values matter for users, the methodology considers a speculative and theoretical approach to value identification. This is particularly concerning given that the role of the context of users greatly influences what values should be targeted, and how these values are defined [14]. Several authors have, therefore, plead for the need for empirical research into the ‘lived value experiences’ of users: a study of values in context [13, 15].

Second, empirical research that studies the actual effect of a novel technology on the values of users is rarely done in VSD. This evokes the ‘positivist problem’, which is the assumption that the embodiment of values in technology directly corresponds with how values are expressed in the use of the technology [8]. VSD, thereby, wrongfully assumes that values are stable constructs [16]. Instead, once a technology is introduced, it starts to ‘mediate’ values. ‘Technological mediation’ refers to the effects of technology on the experiences, actions, and values of users [17, 18]. Value mediation, in particular, refers to the effects of technology on the values of users [19]. A technology can ‘mediate’ or ‘change’ values in multiple ways [16, 20]. Once implemented, it can affect what values are important for users, the definition and meaning given to values (which is also referred to as ‘value dynamism’ [21]), the relative importance of one value over another, the way of translating values into design requirements, and users’ experience of values via norms - “conditions needed to realize values in practice”[20]. All these elements together are termed a ‘value framework’. The basis of VSD is to make design decisions with values in mind. Yet, as these values are mediated once a technology is implemented, the initial design might directly become suboptimal. Therefore, we argue that values cannot be embodied in technology design without anticipating in a real-life controlled experimental context how these values are mediated in the interaction between user, technology, and context.

In this article, we show how empirical research into values can benefit VSD to account for value identification and value mediation. We investigate empirically how a virtual reality (VR) application designed for people with chronic pain is experienced in terms of values. VR is increasingly used in healthcare to manage acute and chronic pain [22–24]. The VR application we studied is designed to enable patients to deal better with their chronic pain daily without the extra burden of pain medication and its side effects, or minimal invasive pain treatments and its possible complications. We will start by empirically studying what and how the values of patients are mediated by experiences of pain. Next, we will empirically study the effects of VR technology and this particular application on patients’ values and provide design and implementation recommendations for more responsible use of therapeutic VR. The article ends with a reflection on the use of this empirical approach contrasting previous, often speculative, literature on values in VR.

## Methods

### Study Design

This study is part of the PijnVRij study (ClinicalTrials.gov Identifier: NCT04042090). The PijnVRij study is a mixed-methods study to assess the effects of therapeutic VR on improving the quality of life in patients with non-specific chronic low-back pain. The quantitative results of the study will be reported separately. Here, we address the qualitative outcomes of the study. The study was conducted from January 2020 till January 2021. Participants were randomized at a pain clinic of a large teaching hospital (Rijnstate, Arnhem, the Netherlands). Approval was obtained under Dutch law by the medical ethical committee (CMO Arnhem-Nijmegen, NL70042.091.19) in compliance with the Declaration of Helsinki.

### Participants

Study participants were adult patients suffering from chronic non-specific low-back pain not attributable to a recognizable, known specific pathology (e.g. infection, tumor, osteoporosis, lumbar spine fracture, or cauda equina syndrome). All participants provided informed consent before participation. Participants were included when they: (1) reported a pain score ≥ 4, (2) did not receive any invasive treatment for non-specific low-back pain in the last year, (3) are willing and able to comply to the study protocol. Participants meeting the following criteria were excluded from the study: (1) participant is included in an alternative trial to evaluate ways of treating pain, (2) participant suffers from severe anxiety, (3) participant has difficulties handling VR (participant has a delirium, dementia, seizure, epilepsy, severe visual/hearing impairment, or head or face is not intact), (4) participant has a high risk of Methicillin-Resistant Staphylococcus Aureus (MRSA).

### Intervention

We studied the VR application Reducept (Reducept, Leeuwarden, The Netherlands). Reducept is a novel psychological VR intervention for treating chronic pain. Reducept aims to teach patients that pain can be influenced and managed by changing how they think about their pain. In the game, the user travels with a spaceship through the body. The journey starts in the spinal cord and ends in the brain. Along the way, the user is educated about the mechanisms of pain. Reducept hereby makes use of the ‘explain pain theory’, which describes that understanding pain and influencing the cognitive, emotional, and behavioral processes related to this pain enables patients to lower their pain sensations [25]. In addition to the pain education, the user plays several serious games stimulating the visual, auditory, and proprioceptive senses to manage chronic pain through distraction and relaxation. The games incorporate the principles of cognitive behavioral therapy, mindfulness, and acceptance and commitment therapy. For example, in one game, the user has to shoot away little ‘insects’ in the body that represent his pain. According to the cognitive behavioral therapy, patients hereby gain control over their pain which can lower the pain experience. In another game, the user is asked to focus on the images visualized in front of him whilst hearing relaxing music. This results in relaxation and mindfulness, which are both beneficial for reduction of a pain sensation. Also, following the acceptance and commitment therapy, users are taught by a relaxing voice that they should accept their pain in their daily lives, which can again reduce the experience of pain [25, 26].

**Fig. 1:**
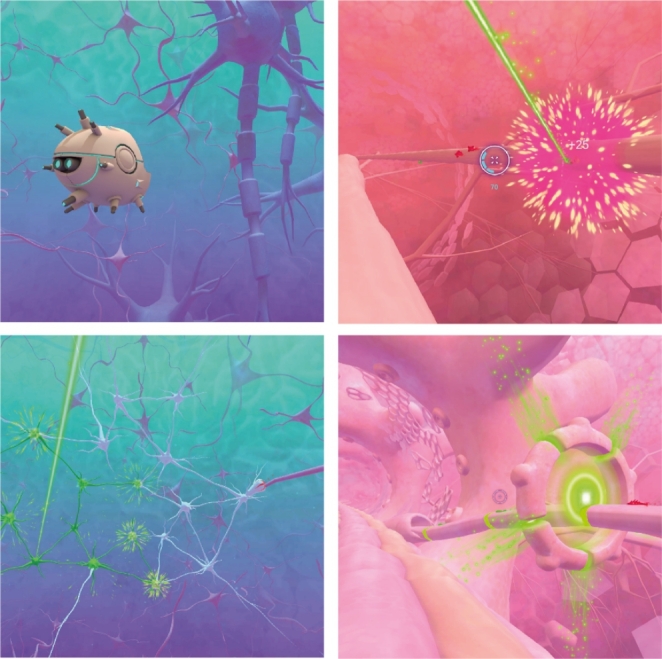
The Reducept application

At the time of the study, the application was still being optimized but a fully working version was available for testing in an experimental controlled setting. Figure 1 portrays screenshots of the application. Reducept was played on the VR headset Oculus Go (Facebook, Inc.; Menlo Park, USA). Participants of our study were inquired to use the application each day at home during a four weeks intervention period. The use of VR and reported pain scores could be monitored through an online dashboard that provided insight into the usage data from a distance.

### Data collection

To comprehend what values patients consider important in the context of their pain, and how VR mediates the values of patients with chronic low-back pain, we interviewed each participant twice, either personally or by phone, using a value-oriented semi-structured interview [27]. The first interview intended to understand the current value framework of patients in the context of chronic pain: what values mattered, the definition of these values, and the effect of pain on each value in terms of norms and experiences. After this interview, participants received education on VR and used the VR application for four weeks. Education was provided to participants in their own homes by a researcher. The researcher explained all buttons and technical features of the VR headset and provided support to connect the headset to the internet. When the participant showed enough knowledge to use the headset autonomously, the researcher left. Participants also received a paper manual with instructions on using VR that repeated the instructions of the researcher. On days 7 and 14, participants received a call from the research team for support when needed. Also, a telephone helpdesk was constantly available when participants needed technical support. The second interview was held directly after four weeks and investigated technological value mediation. We aimed to construct a novel value framework including what values were important in the use of VR, the definition of each value, and patients’ experiences of each value via norms. Both interview guides consisted of open-ended questions and probes derived from literature on important values. Interview guidelines can be found in Appendix A.

### Data analysis

Interviews were recorded and transcribed verbatim. An independent researcher with a background in industrial design and ethics (MS) analyzed all results of the interviews held before using VR separately from al interviews held after using VR. Data were categorized in patient experiences, these into norms, and norms into values. The analysis resulted in two value frameworks. The first value framework consisted of all values patients considered important in the context of their chronic pain before using VR. The second framework refers to the experience of patients after using VR. Value frameworks were then compared to understand how VR technology and this particular application mediates values. Based on this empirical work, the authors then assigned a normative claim to the value mediations. This normative claim is needed to make design decisions. Value mediations were either marked as ‘positive’ (or ‘neutral’), or ‘negative’. Negative value mediations or opportunities for better value mediations were consequently translated into recommendations for the design and implementation of this VR technology. The guidelines for thematic content analysis were followed to ensure study trustworthiness [28].

## Results

### General

**Fig. 2:**
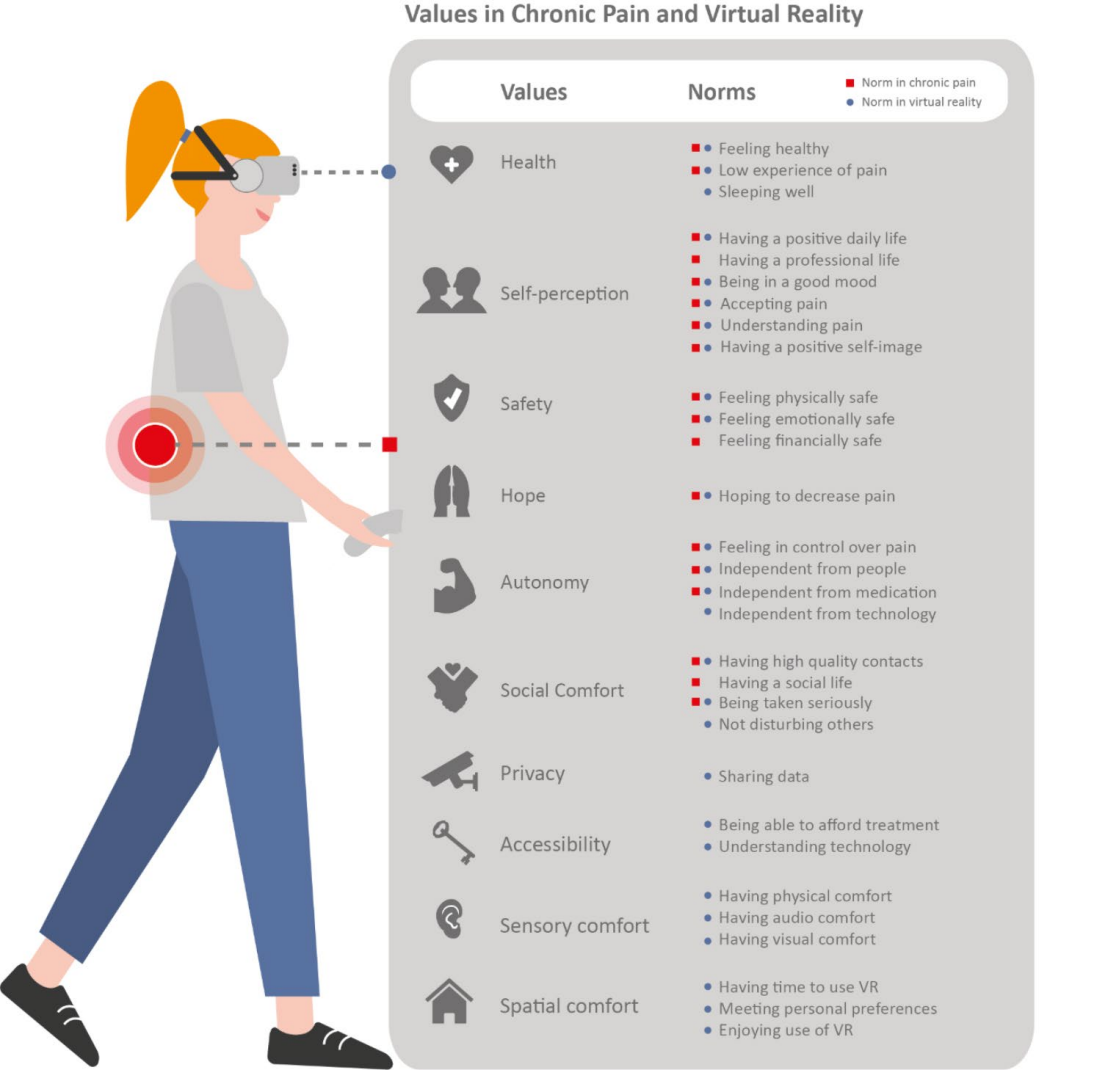
Values in chronic pain and virtual reality

Twenty patients with an average age of 51 (27–71 years old) used VR at home. Seventeen were interviewed before using VR, twenty after using VR. Education levels varied between participants. Three were male, seventeen were female. Only three participants had used a VR headset before. Below, first, the interviews held before using VR are reported. These interviews resulted in six values important in the context of chronic pain: health, self-perception, safety, hope, autonomy, and social comfort. After reporting how these values are defined and expressed in norms and experiences through patient quotes, we report what values matter when VR is used. VR affected the six aforementioned values, and four additional ones were introduced: privacy, accessibility, sensory comfort, and spatial comfort. VR did not affect how people defined the values. Yet, norms related to the values and value experiences were greatly mediated by VR. Recommendations are provided per value to better consider value mediation in this VR design and implementation. Figure 2 visualizes a summary of the results. A more comprehensive overview of the results can be found in Appendix B.

### Value experiences related to non-specific chronic low-back pain

#### Health

All participants experienced chronic pain in the lower back of the body. Pain experience lasted on average already eight years, ranging from one to thirty years. For most, the pain was constantly nagging, intermitted by episodic heavy pain attacks. These were described by terms as: “*stabbing in the back”, “burning”, “shooting nails”*, or *“stinging knives”*. For most, the cause of their pain was unknown. Consequently, most participants had a long history in medical therapy and pain-relieving medication.

Participants were asked to define what it meant for them to feel healthy. First, four participants indicated health to mean feeling well, depending on a healthy food intake, being in a good physical condition, and taking good care of their body. Seven participants defined health as the freedom to be able to live life independently without the constraints of pain. Of them, only four indicated not being healthy due to chronic pain. All others indicated to feel healthy irrespective of the pain. *“The pain does not make me feel unhealthy, it only makes me feel restricted – participant 32 (Female /40 years old)”.* Participants agreed that a life without pain would enable them to more often participate in society, work, undertake activities, exercise, spend time with (grand)children. *“Without pain, I would be able to better enjoy life, without having to constantly fear the pain – 11 (F/35)”*.

#### Self-perception

In the context of chronic pain, this value consists of the norms: activities of daily life, professional life, mood, ability to accept pain, understanding own pain, and self-image. With regards to daily life, for all participants except two pain regularly had profound effects, requiring many to give up sports, hobbies, or daily household chores. Most participants had to consider constantly which activities are possible and which are not. *“I always have to check my shopping basket. When it is too much, I cannot carry it. I always have to consider the pain – 30 (F/71)”.* Moreover, the pain people experienced during short walks hindered multiple participants to leave home. *“I rather stay home all day long, not to feel the pain – 12 (F/50)”.* Pain also influenced participants’ professional life. Five participants indicated to have lost their jobs because of their pain, which remained difficult for them to accept: *“It cannot continue like this. I want to work. I am not the type of person to stay at home – 20 (F/45)”.* Two participants started working fewer hours to handle their pain. Four participants pointed to the importance of continuing their jobs, as it provided them with distraction from pain.

Four participants believed their optimism would not allow pain to affect their mood. All others indicated the pain regularly made them feel unhappy. In periods of heavy pain, they experienced feelings of agitation, frustration, anger, and pessimism. *“After all these years, I have started to become a pessimist – 22 (M/39)”.* Eleven of the participants believed they had accepted the pain as part of their daily lives. They indicated that there is no other way than to accept the pain, as it will not just disappear. Six others had not accepted the pain. *“I do not accept it. I need something to fight for, I need to fight for pain relief – 38 (F/38)”.* Being unable to understand the source of their own pain, negatively affected ten of the participants in the way they looked at themselves. “*I am constantly searching for something that might explain my pain. I cannot accept the pain as long as I have not discovered what is causing my pain – 20 (F/45)*”.

With regards to self-image, all but two participants reflected differently on their bodies due to the pain. For example, six illustrated that they saw their bodies as handicapped: *“Yes, I feel older, I feel handicapped – 15 (F/63)”.* Another six had lost trust in their own body, were disappointed in their own body, felt anger towards their own body, felt vulnerable, or believed their body was failing them. *“I am tired. I hate my body. It hurts everywhere – 20 (F/45)”.* Three felt powerless and incapable to deal with their pain.

#### Safety

The value of safety was divided into the norms: physical safety, financial safety, and emotional safety. Eight participants felt physically unsafe. They illustrated feeling vulnerable, slow in mobility, unable to take care of themselves in case of accidents, and constantly afraid to fall. *“Most people are not aware of my pain. I live alone and I have been thinking lately, what happens if I fall at night? – 30 (F/71)”.* Two participants feared being unable to defend themselves. Only one referenced feeling financially insecure as the pain might force her to give up her job. Concerning emotional safety, six indicated to constantly fear the pain will deteriorate, restricting their freedom. *“I fear that the pain will worsen and that I will not be able to do anything anymore – 15 (F/63)”.*

#### Hope

In this context, all participants hoped to experience pain relief. After years of chronic pain and different treatments, many had not found a solution for pain relief. Four indicated to have lost faith in some of the physicians during the process. Still, most participants kept looking for a solution for solving their pain. Eleven indicated to embrace any treatment offered to reach this aim. *“When you are in pain, you have to try everything – 25 (F/66)”.* Hopes are constantly relatively high at the beginning of a new treatment, oftentimes resulting in disappointment. *“My expectations are very high. I hope so badly he can take away my pain. Or only half of it – 8 (M/53)”*.

#### Autonomy

The value of autonomy was expressed through the norms: feeling in control over pain, independence from people, and independence from medication. All participants felt out of control over their pain. This deficiency of control resulted from painful experiences that cannot be stopped by any means nor predicted on its course. Only small actions as taking medication, doing exercises for the back, finding relaxation, or finding distraction from pain, provided the participants with a minor feeling of temporary control. At the same time, medical specialists had their opinions on how the participants should deal with their chronic pain, and when these did not correspond to the ideas of participants, this led to frustration and dependence. “‘*Oh, madam, you are not allowed to do that! That is not supporting your back.’ But what should I do? Everything hurts my back! I do not feel supported – 30 (F/71)*”. In addition, pain medication made participants feel dependent as eleven expressed worries concerning the side effects of medication. *“I am using pain medication already for years. I am a nurse. I am worried about how the medication is harming my body – 22 (M/39)”.*

#### Social Comfort

Chronic pain affected the social lives of most participants by affecting the quality of contacts, the number of contacts, and being taken seriously by others. Eight participants indicated that the pain made them feel agitated, which directly affected how they responded to the people surrounding them. They frequently felt guilty about how they treated their family in times of pain. In addition, many participants indicated having fewer contacts with others resulting from the pain. *“I have relatively few social contacts, I just do not have the energy – 19 (F/50)”.* Examples were provided on how pain negatively affected social interactions by withholding participants from making appointments, going outside to meet others, playing with grandchildren, or going out with partners. Two participants indicated that losing their jobs isolated them from social contacts even more. Being taken seriously seemed to be an important norm for social comfort. Multiple participants expressed a dislike that others, including treating physicians, did not regularly regard the pain as an important problem. Six participants did not even inform their relatives about their pain. They believed others would not desire to hear their complaints, were afraid others would not believe them, or considered it useless to complain as their relatives would not be able to do anything about it. *“I sometimes think everyone is tired of having me and my pain around. That is why I try to pretend to be healthy – 20 (F/45)”.*

### Value experiences related to the use of VR

Interviews held after participants made use of VR were translated into a novel value framework. Below, we present all values affected by the use of VR in terms of norms and experiences. The same values as discussed in the previous section were found, and four additional ones were introduced: privacy, accessibility, spatial comfort, and sensory comfort. A comparison of the empirical findings of the two value frameworks resulted in VR design and implementation recommendations at the end of each value.

#### Health

Participants were again asked to define this value. Similar definitions were given as in the previous set of interviews. When participants were asked whether VR had affected their feelings of being healthy, all excluding one indicated no effect. This particular participant mentioned pain relief, which provided her with more freedom and a healthier feeling. The remainder of the interviews revealed that other participants also experienced health benefits of VR as well as a new norm, sleeping well.

Half of the participants reported periods with lower pain experience since the use of VR. For three of them, these effects were only felt whilst using the headset as a form of pain distraction. The others reported changes also when not using VR. These mostly resulted from insights the VR brought them regarding their pain. One exercise in the VR game is making the red pain points green. Five participants reported to successfully visualize this exercise when in pain. *“When I am in pain, I think back: make the red ones green. This thought helps to deal with the pain − 12 (F/50)”.* Four other patients had used this technique as well, but unsuccessfully as they could not translate the insights of the game into real life. *“They gave me the exercise to shoot away my pain. But all I think is: how? – 37 (F/44)”.* Although VR affected pain only subtly for most, all participants were positively surprised by these feelings.

Six participants indicated that the VR experience enabled them to find relaxation. Three of them argued that this relaxation lasted long after using the headset. “*I became more relaxed. The VR headset stimulates your mental health – 38 (F/38)”.* One participant explained she lowered her medication intake during the use of the headset. *“Initially, I thought the VR headset did not work for me as I was still in pain. But when I stopped using it, my pain got worse. Besides, I noticed I had started using less medication whilst using the headset – 4 (F/32)”.* Furthermore, weeks after the study, one participant had canceled her planned surgery as the VR had worked for her. Four participants noticed their sleep quality increased due to VR. *“Yes, it is the headset. I used to wake up because of my pain. Now I only wake up when I need to go to the toilet – 16 (F/64)”.* Tiredness was also mentioned twice as a disadvantage of VR.

To conclude, VR seems to positively affect how health is experienced. It added a new norm, sleeping well, to the value, but did not affect the definition of health itself. Yet, results also show that health improvement sometimes remains limited to the virtual world as some users cannot translate virtual games into real-life behavior. To optimize the effects of VR, we recommend:

*A healthy virtual and real me*: *To ensure that VR benefits patients in real life, support is necessary on how virtual lessons could be applied in real-life situations. This support might be incorporated in the VR game or could be provided by caregivers.*

#### Self-perception

This is an important value for people with pain. The previously found norm of professional life was not affected by VR. The others: daily life, mood, accepting pain, understanding pain, and self-image were mediated. One participant believed VR helped her to resume daily life. Another experienced a better mood. Pain acceptance was improved for five. These participants illustrated that VR learned them to accept pain as a part of life, instead of only focusing on the pain. *“The difference is that I used to push away my pain. Now I accept the pain and try to find relaxation – 3 (F/63)”.* Half of the participants became aware of the importance of timely relaxing and not ignoring pain with continuing activities during their busy day. *“I used to be in a constant rush. Never took the time to relax. But now, the headset taught me to relax – 3 (F/63)”.* For one, this insight was emotional: *“The first time I did that exercise, I cried. I was so emotional when I got the insight that I have to find relaxation in my life – 24 (F/52)”.* Half of the participants appreciated the knowledge on the concept of pain they gained by VR. *“It helps to understand what is happening within my body. This knowledge is reassuring – 24 (F/52)”.*

Pain has a strong impact on the norm self-identity. Unfortunately, VR did not positively affect this norm. At a certain moment, the game explained that chronic pain is often caused by the brain, instead of resulting from a physical cause. Five participants could not identify themselves with this image. They even felt offended by the message that their pain was ‘being made up’. Consequently, these participants considered themselves not as the right target audience for this VR application and used this to explain why the headset had not brought them any benefits. *“It annoys me when they say that chronic pain has no specific cause. That the body is not damaged. I thought, my body is heavily damaged. My pain is not an illusion! – 19 (F/50)”.*

To conclude, VR seems to be able to positively mediate the value of self-perception, in particular the ability to accept and understand pain. The norm self-identity was not mediated positively. As this norm is important in patients with pain, we recommend the following:

*Create a positive self-image*: *VR design should make use of the immersive character of VR, enabling patients to embody a positive self-image in real life.*

#### Safety

Norms of physical and emotional safety were mediated by VR. The norm financial safety was not. With regards to physical safety, positive effects were found for three participants who felt stronger after four weeks of using VR. Few adverse effects were reported which negatively affected the norm. Four participants experienced fatigue and dizziness especially the first time. Two were unable to play all games as some games led to pain in the neck. Emotional safety was positively mediated for one patient. She explained that VR taught her to accept her pain and not to fear it constantly. Negative mediation of this norm was found for another person that indicated that the decrease in pain introduced a new fear for her pain to return. Also, full immersion took away awareness of the external environment, which resulted in unsafe feelings for three. That made two of them play VR only when they were alone, doors locked. Contrasting, the third participant only played VR with her radio on, to remain connected to the external world. *“Yes, the first time it scared me. […] I want to play alone, but I turn on the radio in the background. And I play only during daytime. Because it is scary – 30 (F/71)”.* Concluding, VR can positively affect norms related to safety, yet, negative value mediation of safety is also found, mostly regarding full immersion. We recommend:

*Feeling safe and sound*: *VR hardware should allow for a temporary ‘opting-out’ of the virtual environment that enables users to see the real world without taking off the headset to improve their feelings of safety.*

#### Hope

Expectations of participants regarding VR were mediated by many external factors. For some, expectations were magnified by the enthusiasm of medical staff and media attention that the VR experience had generated in the past. Almost half of the participants had seen the VR experience on television or social media before they even had heard of this study. After four weeks of using VR, three participants were consequently heavily disappointed VR had not brought them what they had hoped. “*When I started, I was very positive. I hoped so hard it would help. I believed this would be the solution for solving my pain! […] But it is useless. I am helpless. The pain will last forever– 20 (F/45)”.* Also, as the VR experience has been promoted frequently positively in media, one participant illustrated she began to doubt herself when it did not provide her any benefits. She questioned what she was doing wrong as a reason for why it was not working. *“Many friends had seen this on television. They asked me: are you doing it right? Shit, I thought, I am doing something wrong – 1 (F/61)”.* Other participants did not have high expectations regarding this virtual treatment as they were only waiting for the ‘real’ medical treatment (often surgery) that was already scheduled. As the context of patients affects expectations and resulting outcomes, we recommend:

*The right patient, the right time, the right expectations*: *Medical history and future of patients affect the expectations and hope patients have regarding virtual treatment. The context of a patient should be considered before prescribing VR and whilst supporting patients during VR use to prevent false hopes.*

#### Autonomy

VR added one additional norm to those found previously: independence from technology. More than half of the participants believed to experience more control over their pain after four weeks of using VR. Participants explained they were able to use VR whenever and wherever they desired and did not require the assistance of others in using it. VR was seen as a tool for pain distraction and relaxation. On top of this, it provided participants with the insights that they were able to deal with their pain daily, without simply undergoing it. *“Yes, VR helps me to take control, to take an initiative to deal with my pain. Autonomy over my health, instead of always just waiting for the pain to come – 26 (F/27)”.* Three participants even preferred VR therapy over therapy with a medical doctor, as the gamification demands attention instead of passive listening and, more important, does not judge the participants. At the same time, four participants felt to depend on the technology as it reduced control over the ‘real world’ and demanded to follow the program, instead of doing relaxation exercises at their own pace. To conclude, VR seems to be a promising tool for self-managing health. Self-management, however, does not imply that users should act on their own. All participants greatly valued instructions on the use of the technology before the start and liked the support and encouragement of the research team during use. Therefore, we recommend:

*I can do it!*: *VR improves self-management of pain when patients are well supported on optimal use of the technology to increase their feelings of autonomy.*

#### Social comfort

Social comfort was previously separated in the norms related to quality and quantity of contacts, and being taken seriously. VR affected the norms of quality contacts and being taken seriously and added a norm of not disturbing others. High-quality contact with others was negatively mediated as four participants missed an interaction in the virtual world. Half of the participants desired using the headset only when they were alone as that improved their ability to focus. *“I consciously withdraw myself from my family. I have a place in my office upstairs. It helps me – 22 (M/39)”.* Positive mediation of this norm was found for one participant who believed the headset to have improved her mood, which consequently improved her relationship with her children and spouse. Positive mediations were found for being taken seriously. Almost half of the participants felt the desire to share the VR experience and the corresponding pain education with others. Two participants, finally, greatly disliked disturbing others whilst playing VR. To conclude, our analysis shows that VR design could provide multiple opportunities to become more social:

*VR as a social medium*: *VR provides the opportunity to improve the social contacts of patients. It could allow patients to meet peers, introduce a digital buddy, or allow relatives to empathize with the pain condition of patients.*

#### Privacy

Participants were questioned on their opinion on the use of a dashboard for monitoring VR use from a distance. Various participants directly referred to their privacy, whilst this was not a value found before. Participants compared the dashboard to smart home assistants, a camera in the office, and social media profiles. Yet, none believed their privacy to be harmed. Instead, it was deemed beneficial: making VR more part of the pain treatment, reducing the need to visit the medical doctor personally, and having an external motivator to apply VR on a more regular basis. *“That entire privacy nonsense. You want the doctor to help you. Data exchange is then only beneficial – 22 (M/39)”.* These opinions show us that participants are willing to share personal data to improve their health.

*Do not let privacy harness care*: *Patients should control the ability to exchange their VR usage data and pain experiences with their caregivers to obtain support in their virtual treatment.*

#### Accessibility

Two norms were identified which introduced accessibility as a new value: costs of VR and technical knowledge. In this study, we aimed to make VR accessible: VR headsets were provided for free during the intervention period, and researchers provided support when needed. Nevertheless, accessibility still emerged as an important value. First, two participants desired to continue using VR. Yet, the costs of the headset and the monthly license costs were too high. *“So, I asked the medical doctor, but I had to buy a headset myself. I cannot afford a VR headset, as I am unemployed. Look, I can save some money for buying a headset, but I cannot afford to pay a monthly license – 4 (F/32)”*. Second, three participants indicated that the technical knowledge required to practice VR is a major barrier. Numerous participants referenced technical malfunctions, including a short battery period, inability to charge the device, inability to connect to Wi-Fi, poor vision, and a malfunctioning controller. The research team solved all artifacts. To ensure accessibility of future home-based VR use we recommend:

*Accessible virtual care*: *Any patient should be able to try therapeutic VR. To enable this for any patient, research is necessary on reimbursement and sustainable support services.*

#### Sensory comfort

This value only emerged when VR was used and includes comments on physical comfort, audio comfort, and visual comfort. Concerning physical comfort, thirteen participants had recommendations for improvement. Most disliked that the glasses of the headset quickly became fogged. Additionally, the headset was called heavy, pressing uncomfortably on the skin, pulling down on the head or being too tight on the head, and messing up one’s hair. Two participants were not able to wear their glasses in combination with the headset. One feared the headset would break her glasses: *“I only wore my old glasses, because my current glasses are fragile. I am afraid they would break – 24 (F/52)”*. Concerning the audiovisuals, most participants appreciated what they experienced. Nevertheless, half of them complained about the constant repetition of pain education, which was boring. Also, few participants had blurry sight. We would not regard sensory comfort as a moral value. It can better be seen as a *design* value affecting the willingness to use VR. Therefore, we recommend:

*It’s all in the details*: *All sensory experiences should be considered in the design process to contribute to an optimal and coherent experience. This includes both VR software and hardware.*

#### Spatial comfort

This value could also be seen as a *design* value. This value included all aspects related to comfort participants experience in using VR: having time to use VR, alignment to personal preferences, and joy in using VR. First, the ability participants had in finding a moment for themselves affected the use of VR. Some illustrated they just did not have time to use the headset because of their children or jobs. Others indicated it was difficult to accept they had to sit down during the day. The moment of use, consequently, really depended on the daily schedules of participants. In addition, personal preferences affected use. Nine appreciated continuing using the headset. For some because they liked to do the exercises regularly. Others valued having it at home in case of pain. The final norm, enjoyment, also affected spatial comfort. All participants indicated that using VR for four weeks became boring. Participants agreed on the need for more games, more education, and more competition. To conclude, this value shows how the context and preferences of participants affect willingness to use VR. Possibly, personalization of VR use and software might improve spatial comfort and thereby the other values:

*Personalized technology*: *As each person has different needs, the personalization of software and usage details would optimize virtual treatment.*

## Discussion

We empirically studied in a real-life controlled experimental environment how patients experience VR for treating chronic pain in terms of values. The first set of interviews provided us with insight into what values patients considered important in relation to their pain and illustrated that value definitions and experiences were greatly affected by their daily pain. The second set of interviews illustrated that value frameworks are mediated once VR is used. Although definitions of values did not change, value mediation by VR was found in the list of values important, the translation of the values into norms, and value experiences.

We discuss below per identified value how this empirical approach to VSD compares to previous, often speculative, research into values in VR. The comparison highlights that empirical research actually provides valuable insights that cannot be anticipated theoretically. We illustrate the advantages and disadvantages of empirical research contrasting a speculative, theoretical approach. First, in contrast to speculation about values, empirical research results in many more moral *goods* found in VR than moral *concerns*. This increases the acceptability of a technology. Second, empirical research provides context- and even person-specific insights, whilst a speculative approach mostly creates general insights. The specific insights enable for easier translation of values into design requirements, and even more, personalizing technology to individual users. Being so context-specific is also a disadvantage as it disables generalizing insights to other application domains. Third, empirical research enables to identify and define values that users consider important in context whilst these might not have been identified in speculation. However, this is also a risk of empirical research. It only shows values users consider important, whilst there might be values that need consideration nonetheless. Speculative anticipatory reflections, therefore, might still be necessary in addition to empirical research. Fourth, being able to identify value mediation in practice, enables for a redesign of the technology and its way of implementation to ensure that initial value embodiment corresponds with how values are actually expressed in practice. A disadvantage of empirical research is that it is time-consuming to conduct empirical research. The time and resources for research and ability to study a prototype of a technology in a controlled experimental environment might not always be available to development teams. Each VSD development team should weigh the advantages and disadvantages of empirical research. When possible, empirical research is generally preferred over speculation only.

### Health

In contrast to the other values, ‘health’ has been studied empirically often. Multiple studies have been conducted in the medical domain on the effectiveness of VR for health. Our study results are in line with those of Snelgrove, Edwards [29], showing chronic pain to be constantly affecting patients’ well-being. Interviews held after using VR suggest that VR can improve health. These findings comply with findings from studies on improving pain management skills by VR [22, 30–32]. To even better deal with pain in real life, we recommended the provision of support to patients to translate virtual lessons to real life. Games that support changing real-life user behavior have been described as *persuasive games* [33]. Therapeutic VR could consider principles of persuasive game design to optimize healthy patient behavior in real life.

### Self-perception

Several authors have speculated on the ability of VR to alter the self-perception of individuals [34, 35]. They stated that because VR can create ‘virtually real experiences’ [36], users of VR quickly identify themselves with the virtual arms or virtual heartbeat that they can hear [37–39]. This illusion of embodiment could result in loss of self-identity by alienating users from their own body, and a loss of connection to the real world, which could negatively affect behavior in real life [40]. In the VR application we tested, this ability to change the way people phenomenologically experience their bodies is intentionally used to lower experiences of chronic pain. Before using VR, most participants expressed that chronic pain has altered the way they perceive themselves, which complies with prior research [41]. After using VR, several participants positively experienced a change in self-perception; most participants indicated to accept pain better as part of daily life and to comprehend the concept of pain better. Our findings did not correspond with the speculative concerns. It was in this context that Tack [42] posed the concern of VR providing a temporary illusion of a ‘stronger’ back. He questioned whether this would cause patients to act beyond their physical capacity and if the return to self-perception would lead to disappointment. Again, our findings showed differently. In leaving VR, our participants did not experience disappointment. Even better, some were able to apply lessons learned from the VR application in real life. By, for example, ‘shooting away’ their pain, patients could embody that ‘stronger’ back. This might result from cognitive-behavioral therapeutic principles, including acceptance and commitment therapy and mindfulness, underlying the VR game [25, 43] which is a promising tool for any therapeutic VR to increase patient empowerment. This value particularly shows that empirical research into values provides more positive results than speculation.

### Safety

Participants commented that their experiences of chronic pain often negatively affected their feelings of physical safety. Previously, Horsfield [40] illustrated that the virtual world could enable people to experience greater safety by escaping from pain and mortality as these are part of our embodiment. We found that VR indeed allows for pain distraction and thereby temporarily increased feelings of safety in a virtual world. Horsfield continued that temporary feelings of safety in the virtual world could lead to alienation and disappointment in the real world. We have not found this to be true. Actually, virtual feelings of safety were embodied by three participants, that consequently felt safer in the real world. In contrast, virtual environments could also harm safety by causing adverse effects like motion sickness [44]. In our study, only a few patients experienced minor dizziness after using VR, as the VR application was designed to prevent this. We did find another negative effect of VR on safety though. Especially the first time, some participants felt insecure in the virtual environment due to its novelty and being disclosed from the real world. These empirical findings generate other insights than speculative claims and should be considered by any therapeutic VR team during design and implementation.

### Hope

Horsfield [40] speculated that the virtual world is a place of hope. It can provide hope that daily life could be lived without pain. The author continued that this could lead to disappointment and disillusion. Our research provided nuance to this claim. In the study, the experience of VR itself did not directly mediate hope. It was the medical condition of patients that initially created a boundless hope that pain one day will disappear. VR became the object of hope. Bolstered by advertisements, media attention, and enthusiasm of the medical staff, this hope might have generated false beliefs and affected the actual impact of VR. Our findings are in line with a concern earlier on posed by Madary and Metzinger [35] called ‘therapeutic misconception’. They argued that VR, as a novel technology, generates high expectations when applied in healthcare. As these could result in desperation and disappointment, a shared decision-making process between patient and caregiver in which realistic expectations are discussed was recommended before prescribing therapeutic VR.

### Autonomy

In literature, speculative concerns have been raised on the dependence of patients on VR [34]. We did not find a dependence on VR but only found that VR offers potential for self-management and increased feelings of autonomy. Nevertheless, we recommended that support is still necessary to allow any patient to make use of the technology. This recommendation complies with previous recommendations on support needed in ehealth use to foster autonomy [45].

### Social Comfort

Concerns in the literature addressing social relationships, including escapism from the real world [46], were not encountered in our study. Yet, we found that VR is not contributing to social relations either. We also identified an opportunity for VR to become more social. Previously, it has been concluded that VR can contribute to social relations and empathy [47, 48]. Within healthcare, VR can be used in this way by improving empathy for people with dementia in caregivers [49, 50] and by using the virtual environment as a ‘playground’ for hospitalized children [51]. These cases illustrate that social comfort is a value that could be improved by VR and might become incorporated in any therapeutic VR design.

### Privacy

Our findings contrast common literature regarding privacy as one of the most pressing ethical concerns of VR. Madary and Metzinger [35], O’Brolcháin, Jacquemard [52], and Spiegel [53], for example, speculated on the value of privacy. The authors feared for people’s privacy when eye movement is traced, data is being stolen or misused, and third parties obtain access to VR’s camera to record faces. Privacy was not seen as an issue by patients. Even though our patients did not consider these concerns, designers should make sure these items never become patient concerns. Therefore, we recommended VR developers to consider, amongst others, general data protection regulation guidelines, and the use of VR headsets designed for medical purposes. This way, privacy will not constrain data sharing between patient and caregiver for better support. This value shows that empirical research might not provide a full insight into all values of importance, as users might not always know what is best for them.

### Accessibility

Access to VR is repeatedly speculated as an ethical concern [35, 40]. Our results correspond with this concern. We made VR available to patients by offering them VR headsets for free and providing education and technical support. Still, these aspects remained a barrier for accessibility as some desired continuing use of VR and encountered financial problems, and others pointed to technology as a barrier for future use. To achieve distributive justice in virtual care technology, there is a need for insight into the cost-effectiveness of VR therapy compared to traditional therapy so that medical insurance agencies are willing to reimburse. Furthermore, we recommended education and support services to make therapeutic VR accessible for all.

### Comfort

Technology development is oftentimes steered by the possibilities that technology offers. Yet, these possibilities do not always meet the needs of users. Therefore, Kellmeyer [34] argued that instead of opting for a technology-centered approach, VR development should follow a user-centered approach. Our study demonstrated the importance of this statement through the values of sensory and spatial comfort. We labeled these values *design* values because they do not directly relate to moral considerations. We encouraged both ethicists and designers to consider these values together with moral values to optimize the mediation of VR.

### Limitations

This study has a few limitations. First, as the VR experience was new to all but three participants, this novelty might have influenced, for example, positive outcomes related to health, negative experiences of safety, and high expectations and hope. Repetition of this study within some years, when VR is a well-known technology, will clarify whether the novelty of this technology has affected the study outcomes or whether outcomes are intrinsic to VR. Second, during the study, the Reducept application was updated three times with minor changes. The updates went together with several technological failures. These have affected values of sensory and spatial comfort. Ideally, studies into (VR) software should be conducted via only testing one version. Third, we studied participants with non-specific chronic low-back pain awaiting medical treatment. Our study results reflect their opinions and might differ from people suffering from different types of chronic pain, from patients not following a trajectory for another treatment, or from patients with a shorter history of pain, especially regarding the values of health and hope. Only with a longitudinal study in a larger patient population, we would be able to generalize our value results to chronic pain and VR. Fourth, as we applied semi-structured value-oriented interviews, we had identified through literature a set of values of which we believed pain and VR might affect. Although we provided the interviewees the opportunity to come up with topics themselves and to only continue with the topics they deemed most important, this technique of interviewing might have influenced the outcomes of the value identification process. Also, the normative process of translating empirical research into design and implementation recommendations remains a subjective endeavor. Nonetheless, to empirically identify values, and appreciate value experiences, no better methods exist at present. Fifth, we did not study the relative importance of one value over another even though this is part of a value framework [16, 20]. This relative importance is particularly important in the case of value tensions. When these occur, insight into users’ value prioritization could aid in solving the tensions [54]. Finally, this study has been conducted during the COVID-19 pandemic. This pandemic required people to remain at home and decreased their mobility. Several participants questioned whether their experiences were also affected by changes in social contact and stress. This observation stresses the importance of taking into account both micro and macro contextual factors when studying the value experiences of people.

## Conclusions

In this article, we studied the added benefit of an empirical approach to VSD to inform value identification and anticipate value mediation. We investigated the effects of VR on patient values by analysis of the patient-technology interaction in the context of chronic pain. Insights were translated into normative recommendations for a values-based design and implementation of VR treatment. We started this article by illustrating that VSD remains vague about the role of empirical research in its approach. It relies on speculation to identify values and assumes that value embodiment results in equal value expression when the technology is applied in its context. The case study showed the advantages of empirical research contrasting mere speculation on values. It enables a more ‘positive’ view on values, provides detailed context-specific insights required to align design with users’ values, can identify a list of values based on what users consider important, and enables anticipating value mediation in the design process. Nonetheless, a speculative approach to values might still be needed in specific situations. For example, when the technology is not available for study, or when users cannot account for all values of importance. In addition, empirical research is time-consuming, and results can only be generalized to similar application domains. When possible, VSD should include empirical research for value identification and anticipation of value mediation in its process to optimally and responsibly inform technology design.

## Electronic supplementary material

Below is the link to the electronic supplementary material.

Supplementa﻿ry Material 1

Supplementary Material 2

## Data Availability

The datasets generated during and/or analysed during the current study are available from the corresponding author on reasonable request.
