# Supplementary material for: Evaluating value mediation in patients with chronic low-back pain using virtual reality: contributions for empirical research in Value Sensitive Design
Source: Health Technol (Berl). 2022 Apr 29;12(4):765–78. doi: 10.1007/s12553-022-00671-w (PMC9051502; doi:10.1007/s12553-022-00671-w)
Supplement: Supplementary file 1 — Supplementa﻿ry Material 1 [file 12553_2022_671_MOESM1_ESM.docx]

**Evaluating value mediation in patients with chronic low-back pain using virtual reality: contributions for empirical research in Value Sensitive Design**

**Health and Technology**

Merlijn Smits, MSc., EDAC.

Harry van Goor, MD, PROF., PhD, FRCS.

Jan-Willem Kallewaard, PhD, FIPP.

Peter-Paul Verbeek, PROF., PhD, IR.

Geke D.S. Ludden, PROF., PhD, IR.

**Corresponding author:** Merlijn Smits, MSc, EDAC, Department of Surgery, Radboud University Medical Center, P.O. Box 9101, 116, Geert Grooteplein Zuid 10, 6525 GA Nijmegen, the Netherlands, Email: [Merlijn.Smits@radboudumc.nl](mailto:Merlijn.Smits@radboudumc.nl), +31243617612

**Appendix A: Value-oriented semi-structured interview guidelines**

**Interview guide before using VR**

1. Could you describe your feelings of pain?
2. Do you understand the cause of your pain?
3. What is affecting your pain?
4. Is there something you can do to relief your pain?
5. Did the pain change something for you in your life?
6. Does the pain affect how you consider yourself?
7. Does your experience of pain affect your feelings of safety?
8. Does your experience of pain affect your social life?
9. Do you feel healthy?
10. What are your expectations regarding the use of VR?

**Interview guide after using VR**

1. What did you think of using VR?
2. Where and when did you use VR?
3. How did you appreciate the use of a VR dashboard?
4. Did VR change something for you?
5. Did VR affect your experience of pain?
6. Did VR affect how you perceive yourself?
7. Did VR affect your relations with other people?
8. Did VR affect your feelings of being healthy?
9. Did VR affect your feelings of autonomy?
10. Did VR affect your feelings of safety?
11. How do you rate the comfort of the VR experience?
12. What would be your ideal VR experience?
13. Would you want to use VR in the future as a tool for pain relief?

**Appendix B. Value mediation of patients with non-specific chronic low back pain using VR**

|  | | **Interview series 1 (n)** | **Interview series 2 (n)** |
| --- | --- | --- | --- |
|  |  | Health =  Having no pain  Freedom to act according to own preferences  Having a positive mindset and a healthy lifestyle | |
| **Health** | **Norm** | **Value mediation chronic pain (n)** | **Value mediation VR (n)** |
|  | Feeling healthy |  | |
|  |  | + Feeling healthy even when in pain (10) | + VR lowered pain and therefore improved feelings of health (1) |
|  |  |  | |
|  |  | - Not being able to feel healthy due to the pain (4) | - VR did not affect feeling healthy (19) |
|  | Having a low experience of pain |  | |
|  |  | *Relaxation*  + Relaxation lowers pain experience (12)  *Distraction*  + Distraction lowers pain experience (4)  *Medication*  + Medication lowers pain experience (13)  *Physical exercise*  + Physical exercise lowers pain experience (8)  *Other*  + Warmth lowers pain experience (2)  + Transcutaneous Electrical Nerve Stimulation (TENS) lowers pain experience (4) | *Relaxation*  + VR is a tool for relaxation during its use (6)  + VR is a tool for relaxation after its use (3)  *Distraction*  + VR allowed to distract from pain during its use (4)  *Medication*  + VR allowed for lowering medication intake (1)  *Pain experience*  + VR lowered pain experience after its use (7)  + VR lowered pain experience during its use (3)  *Future treatment*  + No need for invasive treatment after VR (1) |
|  |  |  | |
|  |  | *Pain experience*  - Specialists have not found a solution for pain relief (17)  - Being in a constant pain (16)  - Experiencing sporadic pain attacks (1)  *Medication*  - Medication intake is too high (11) | *Pain experience*  - VR does not affect pain experience (9)  - Pain experience got worse during period of using VR (2)  - Pain has changed in location during period of using VR (1)  *Medication*  - VR does not affect medication intake (19)  *Relaxation*  - Being too stressed to use VR as a tool for relaxation (3)  *Future treatment*  - VR does not affect future treatment (19) |
|  | Sleeping well |  |  |
|  |  |  | + VR improved sleep quality (4) |
|  |  |  |  |
|  |  |  | - Using VR resulted in sleepiness (2) |

|  | | **Interview series 1 (n)** | **Interview series 2 (n)** |
| --- | --- | --- | --- |
|  |  | Self-perception=  Understanding the self and all aspects to it | |
| **Self-perception** | **Norm** | **Value mediation chronic pain (n)** | **Value mediation VR (n)** |
|  | Having a positive daily life |  | |
|  |  | + Pain does not affect daily life (2) | + VR provided optimism to resume daily life activities (1) |
|  |  |  | |
|  |  | - Having to consider pain in every part of daily life (14)  - Pain decreases ability to leave home (5) |  |
|  | Having a professional life |  | |
|  |  | + Pain does not affect work (10) |  |
|  |  |  | |
|  |  | - Participant is unable to work because of pain (5)  - Pain influences how work is being done (2) |  |
|  | Being in a good mood |  |  |
|  |  | + Pain does not affect mood (4) | + VR improved mood (1) |
|  |  |  |  |
|  |  | - Pain negatively affects mood (8) |  |
|  | Accepting pain |  |  |
|  |  | + Being able to accept the pain (11) | + VR improved knowledge on need for relaxation (8)  + VR allowed for better pain acceptance (5) |
|  |  |  |  |
|  |  | - Not being able to accept pain as part of life as it affects life too much (4)  - Not being able to accept pain as the cause is unknown (1)  - Not being able to accept pain as that would result in loss of a reason to fight for pain relief (1) |  |
|  | Under-standing pain |  | |
|  |  |  | + VR improved knowledge on pain (10) |
|  |  |  | |
|  |  | - No cause for pain is known (10) | - VR did not improve knowledge on pain (4) |
|  | Having a positive  self-image |  | |
|  |  | + Pain has not affected self-image (3) |  |
|  |  |  | |
|  |  | - Pain makes participant feel handicapped and old (6)  - Trust in body is gone because of pain (6)  - Pain makes participant feel powerless (3)  - Pain has affected the appearance of the body (2)  - Pain causes self-pity (1)  - Participant hates body because of pain (1)  - Participant feels minor because of pain (1) | - Message of “pain is in the head” is frustrating (5) |

|  | | **Interview series 1 (n)** | **Interview series 2 (n)** |
| --- | --- | --- | --- |
|  |  | Safety =  Being resilient and strong  Financial security in life | |
| **Safety** | **Norm** | **Value mediation chronic pain (n)** | **Value mediation VR (n)** |
|  | Feeling physically safe |  | |
|  |  | + Feelings of physical safety are not affected by pain (6) | + VR improved feelings of safety by reducing pain (3) |
|  |  |  | |
|  |  | - Feeling insecure because of the fragile body (6)  - Being unable to defend yourself (2)  - Having no feelings of physical safety at all because of pain (1)  - Feeling insecure because of the loss of flexibility in the body (1)  - Being unable to take care of yourself in case of accidents (1) | - VR has not affected feelings of physical safety (8)  *Adverse effects*  - Dizziness after playing VR (4)  - VR is not suitable to use during a headache (3)  - Fatigue after playing VR (2)  - VR created a pain in the neck (2) |
|  | Feeling financially safe |  | |
|  |  |  |  |
|  |  |  | |
|  |  | - Pain creates a fear for financial safety (1) |  |
|  | Feeling emotionally safe |  |  |
|  |  |  | + VR reduced the fear for pain (1) |
|  |  |  |  |
|  |  | - Being in constant fear that pain will worsen (4)  - Fearing surgery as it could affect own freedom (2) | - As VR reduces feelings of pain, participant fears the pain to return (1)  - VR shuts off participants from real world, this created feelings of unsafety (3) |

|  | | **Interview series 1 (n)** | **Interview series 2 (n)** |
| --- | --- | --- | --- |
|  |  | Hope=  Having a positive expectation towards the future | |
| **Hope** | **Norm** | **Value mediation chronic pain (n)** | **Value mediation VR (n)** |
|  | Hoping to decrease pain experience |  | |
|  |  | + Participants believe their pain experience can be reduced in the future (11) | + Participants believed VR will bring them small benefits (9) |
|  |  |  | |
|  |  | - Having lost faith in therapists (4)  - Participants do not get their hopes up anymore to prevent disappointment (4)  - Costs of treatment reduce prospect of pain treatment (3)  - Waiting time of treatment reduce prospect of good pain treatment (1) | - Media attention might generate false hope (7)  - Participants were disappointed after false hopes (3)  - Therapists might generate false hope (3)  - Participant feared side effects of VR (1)  - Participant feared pain is too heavy for VR (1)  - As VR did not reduce pain, participant started to doubt herself (1) |

|  | | **Interview series 1 (n)** | **Interview series 2 (n)** |
| --- | --- | --- | --- |
|  |  | Autonomy=  Being able to independently live life | |
| **Autonomy** | **Norm** | **Value mediation chronic pain (n)** | **Value mediation VR (n)** |
|  | Feeling in control over pain |  | |
|  |  | + Small actions allow temporarily for lower pain experiences (4) | + VR allowed for better control over pain (11) |
|  |  |  | |
|  |  | - No feelings of control over pain (12) | - VR did not allow for better control over pain as no pain relief was experienced (3) |
|  | Being independent from people |  | |
|  |  |  | + VR does not judge (3) |
|  |  |  | |
|  |  | - Not agreeing with advice of medical doctors (4) |  |
|  | Being independent from medication |  |  |
|  |  |  | + VR allowed to lower medication intake (1) |
|  |  |  |  |
|  |  | - Relying heavily on medication intake (11)  - Having to lower medication intake against own will (1) |  |
|  | Being independent from technology |  | |
|  |  |  |  |
|  |  |  | |
|  |  |  | - Reduced control over the real world (3)  - Feeling dependent on the speed and program in VR (1)  - Being unable to purchase VR whilst it is offering benefits (1) |

|  | | **Interview series 1 (n)** | **Interview series 2 (n)** |
| --- | --- | --- | --- |
|  |  | Social comfort=  Having comfortable social relations | |
| **Social Comfort** | **Norm** | **Value mediation chronic pain (n)** | **Value mediation VR (n)** |
|  | Having high quality contacts with others |  | |
|  |  | + Pain does not affect relations (5) | + VR improved mood, which improved relation with others (1) |
|  |  |  | |
|  |  | - Reacting irritated to other people due to the pain (8) | - VR is preferably done alone (10)  - VR is not interactive (4) |
|  | Having a social life |  | |
|  |  | + Meeting people in professional life (2) |  |
|  |  |  | |
|  |  | - Having a very small social life due to the pain (9)  - Being isolated at home after losing a job (2) |  |
|  | Being taken seriously |  |  |
|  |  |  | + Desire to share VR experience with others (7) |
|  |  |  |  |
|  |  | - Pain is kept a secret for others (6)  - Pain is not being taken seriously by others (5) |  |
|  | Not disturbing others |  |  |
|  |  |  |  |
|  |  |  |  |
|  |  |  | - Using VR disturbed others in the room (2) |

|  | | **Interview series 1 (n)** | **Interview series 2 (n)** |
| --- | --- | --- | --- |
|  |  |  | Privacy=  Disclosure of personal data |
| **Privacy** | **Norm** | **Value mediation chronic pain (n)** | **Value mediation VR (n)** |
|  | Sharing data |  | |
|  |  |  | + VR should improve own well-being; a dashboard does not affect that (5)  + The dashboard for therapists improved motivation (2)  + The dashboard improved involvement of medical doctor (2)  + The dashboard might reduce the need to visit the medical doctor personally (1) |
|  |  |  | |
|  |  |  |  |
|  | | **Interview series 1 (n)** | **Interview series 2 (n)** |
|  |  |  | Accessibility=  Availability for everyone |
| **Accessibility** | **Norm** | **Value mediation chronic pain (n)** | **Value mediation VR (n)** |
|  | Being able to afford treatment |  | |
|  |  |  |  |
|  |  |  | |
|  |  |  | - VR headset and license are expensive (2) |
|  | Under-  standing technology |  | |
|  |  |  | + VR was easy to use (6) |
|  |  |  | |
|  |  |  | - It was difficult to focus in VR (5)  - VR had too low battery capacity (5)  - VR was a difficult technology (3)  - The pointer broke down (3)  - VR software not working properly (1)  - VR did not want to connect to Wi-Fi (1)  - VR did not charge (1) |

|  | | **Interview series 1 (n)** | **Interview series 2 (n)** |
| --- | --- | --- | --- |
|  |  |  | Sensory comfort=  Comfortable sensory experiences |
| **Sensory Comfort** | **Norm** | **Value mediation chronic pain (n)** | **Value mediation VR (n)** |
|  | Having physical comfort |  | |
|  |  |  | + VR was comfortable (4) |
|  |  |  | |
|  |  |  | - Glasses of headset got foggy (5)  - Headset was too tight or too loose (5)  - Headset was too heavy (4)  - Headset was pressing in the face (2)  - Fear that headset might break own glasses (1)  - Headset does not fit over own glasses (1)  - Headset messed up haircut (1) |
|  | Having audio comfort |  | |
|  |  |  | + Sound was fun (1) |
|  |  |  | |
|  |  |  | - Constant repetition of education is boring (8)  - Volume button could not be found (1)  - VR should be connected to a headphone (1)  - Preference for personalization of music (1) |
|  | Having visual comfort |  |  |
|  |  |  | + Graphics were nice (4) |
|  |  |  |  |
|  |  |  | - Being unable to obtain a visual focus (4) |

|  | | **Interview series 1 (n)** | **Interview series 2 (n)** |
| --- | --- | --- | --- |
|  |  |  | Spatial comfort=  Feeling comfortable in using the technology |
| **Spatial Comfort** | **Norm** | **Value mediation chronic pain (n)** | **Value mediation VR (n)** |
|  | Having time to use VR |  | |
|  |  |  |  |
|  |  |  | |
|  |  |  | - It is not easy to make time to use VR (10) |
|  | Meeting personal preferences |  | |
|  |  |  | + Being able to use VR only as a distraction from current pain (6)  + Being able to use VR on a regular basis (6) |
|  |  |  | |
|  |  |  |  |
|  | Enjoying use of VR |  |  |
|  |  |  | + Immersion is unique in VR (6) |
|  |  |  |  |
|  |  |  | - VR became boring after some weeks (14)  - VR does not offer enough content (7)  - Purpose of VR can also be reached by 2d visuals (1)  - Relaxation works better without visuals (1) |
